# Evaluating the Effectiveness of Interventions on Increasing Participation in Cervical Cancer Screening

**DOI:** 10.1097/jnr.0000000000000317

**Published:** 2019-09-20

**Authors:** Gonul KURT, Aygul AKYUZ

**Affiliations:** 1 PhD, RN, Assistant Professor, Faculty of Health Sciences, Department of Midwifery, Sakarya University, Sakarya, Turkey; 2 PhD, RN, Professor, Department of Obstetrics and Gynecology Nursing, Florence Nightingale Hospital School of Nursing, Demiroglu Bilim University, Istanbul, Turkey.

**Keywords:** cervical cancer screening, home visit, invitation, cervical cancer, Pap smear test

## Abstract

**Background:**

Although cervical cancer is highly preventable through regular screenings using Pap smear or human papillomavirus–deoxyribonucleic acid tests, cervical cancer remains a prevalent women's health issue across the world. Therefore, encouraging women to screen for cervical cancer is very important for the early detection of cervical cancer.

**Purpose:**

The purposes of this study were to (1) assess the effectiveness of three interventions that are typically used to increase the uptake of cervical cancer screening during home visits and (2) determine the participation rate in cervical cancer screenings after invitation, the health promotion perceptions, and the cervical cancer and screening-related knowledge of women. The three interventions noted in Purpose 1 were one-on-one training accompanied by an educational brochure, providing the educational brochure only, and giving an invitation without any relevant information.

**Methods:**

This interventional study was conducted on women who were between the ages of 30 and 65 years in three Turkish provinces (Ankara, Malatya, and Trabzon). Five hundred twenty home visits were made, and 356 women who did not have a Pap smear test within the previous year were invited for cervical cancer screening. Women were randomized into one of three intervention groups, and the participants in each group were invited to attend a national cervical cancer screening program and to undergo a cervical cancer screening using the related intervention type.

**Results:**

The results showed that the interventions used during home visits and knowledge were effective in encouraging women to participate in cervical cancer screening. It was determined that the participants who had received one-on-one training accompanied by an educational brochure had a higher cervical cancer screening rate than their peers who were offered a brochure only or a verbal invitation only.

**Conclusions:**

Invitations to screenings that are made by providing training accompanied with a brochure were found to be effective in increasing the participation of women in cervical cancer screening.

## Introduction

Cervical cancer is the second leading cause of cancer mortality among women worldwide, with approximately 530,000 new cases of invasive cervical carcinoma and approximately 280,000 related deaths reported each year. About 95% of the cases occur in less developed countries. The incidence of this disease is reportedly increasing, particularly in developing countries (Globocan, 2018; Visanuyothin, Chompikul, & Mongkolchati, 2015; World Health Organization [WHO], 2018). In Turkey, cervical cancer is the third most common type of cancer among gynecologic malignancies, with an incidence of 4.5 cases per 100,000 (Public Health Institute of Turkey, Cancer Control Center, 2018). According to Globocan (2018), in Turkey, an estimated 2,733 new cases will be found and approximately 1,553 related deaths will occur because of cervical cancer by 2025 (Globocan, 2018).

Cervical cancer is highly preventable through regular and appropriate, timely screenings (Bassal, Schejter, Cohen, & Keinan-Boker, 2017; Visanuyothin et al., 2015). Thus, cervical cancer is included among the cancers for which screening programs are recommended by the WHO. The most common technique recommended by WHO for the screening and early diagnosis of this disease is Pap smear or human papillomavirus–deoxyribonucleic acid tests (American Cancer Society, 2015; Gücük, Alkan, Arica, & Ateş, 2011; Lu, 2014; Wright & Kuhn, 2012). Cervical cancer screening (CCS) programs with Pap smear or human papillomavirus–deoxyribonucleic acid tests have been shown to decrease the incidence of cervical cancer by 34%–80% and the related mortality rate in many developed countries (DeGregorio et al., 2017; Kitchener et al., 2018; Visanuyothin et al., 2015). A wide variety of Pap smear tests and uptake of CCS procedures exist around the world. According to the results of studies conducted in various countries, the ratio of women who have received at least one CCS with Pap smear test varies widely, with rates of 52.6% in Taiwan, 66% in Mexico, 72% in Botswana, 93% in the United States, 30.5% in Malaysia, 23.1% in China, 5.3% in India, and 4.1% in Morocco (Gakidou, Nordhagen, & Obermeyer, 2008; Lee & Wang, 2011; Lee-Lin et al., 2007; Mingo et al., 2012). The CCS participation rate in domestic Cancer Early Diagnosis, Screening and Education Centers (KETEMs), which are the only facilities that conduct community-based cancer screening in Turkey, was reported to be only 11.3% in 2008 (Özgül, 2010; Tuncer, Özgül, Özen Olcayto, Gültekin, & Erdin, 2009). Studies conducted in various regions and on various groups in Turkey have also reported low participation rates for CCS (varying between 11% and 56%; Babacan Gümüş & Çam, 2011; Güvenç, Akyuz, & Yenen, 2013; Uluocak & Bekar, 2012). These results show that the participation rate for CCS in Turkey remains below desired levels and below the levels that are prevalent in developed countries.

The participation of women in CCS is known to be inadequate in many developing countries. The sociodemographic and cultural characteristics of the women, health beliefs, their knowledge about cervical cancer and the Pap smear test, and the lack of available screening programs were all found to influence the participation rates of women in CCS (Güvenç et al., 2013; Lee, 2018; Lu, 2014; Uluocak & Bekar, 2012). In different areas and in light of the results of various studies across Turkey, reasons why women do not participate in CCS include lack of knowledge about CCS and national screening program services, health beliefs (i.e., believing CCS is not necessary if there are no symptoms, ignoring the necessity of CCS, and fear that the Pap smear test is painful), perceived barriers related to CCS (i.e., fear of results and embarrassment), and environmental factors (i.e., difficulties in accessing healthcare units; cost, quality, and continuation of the service; and long waits at the healthcare center; Babacan Gümüş & Çam, 2011; Güvenç et al., 2013; Uluocak & Bekar, 2012; Yücel, Çeber, & Özentürk, 2009). In addition, sociocultural norms and religious beliefs affect the willingness of women in Turkey to participate in CCS. CCS-related examinations cause embarrassment for women, who prefer that a female physician conduct the procedure. One study conducted in Turkey reported that the women who had never undergone a Pap smear test had significantly higher perceived barriers to CCS than women who had. The same study reported that gynecologic-examination-related embarrassment and preference for a female physician affected women's decisions to participate in CCS (Güvenç et al., 2013). Similarly, another study showed that women who found the Pap smear test painful and did not know where they could go to have a Pap smear test were more likely to avoid having this test (Esin, Bulduk, & Ardic, 2011). As these results show, it may be argued that the most important determinants of participation in CCS are knowledge, awareness, and perceived barriers about this subject. Several studies have noted that removing the perceived barriers to and fostering awareness of cervical cancer, risk factors, and the CCS program, as well as using interventions to provide information about CCS and to develop health protection behaviors, have all been reported to be important steps in increasing the participation of women in CCS programs (Boonpongmanee & Jittanoon, 2007; Güvenç et al., 2013; Kitchener et al., 2018; Lu, 2014). Bowles, Gao, Brandzel, Bradford, and Buist (2016) reported that sending out Pap-specific letters containing reminders of the availability of multiple preventive services was effective at promoting CCS (Bowles et al., 2016). In a study that was conducted to increase participation in CCS, 200 women were given one-on-one education and 200 were trained using a video produced by a national television channel. They found the 3-month posttest screening rate (after the one-on-one education) was higher in women who were educated using the video (Lam et al., 2003).

Nurses play a critical role in providing preventive healthcare services. Moreover, nurses who are trained in early diagnosis cancer programs in developed countries play an essential role in public health screenings and educational programs by cooperating with other healthcare professionals (Anttila & Nieminen, 2007; Güvenç et al., 2013; Vokó et al., 2012). The responsibility of nurses include meeting the information needs of women by giving them healthcare training, encouraging women to participate in screening, implementing Pap smear tests, collecting and evaluating screening data, and directing women to healthcare services for early diagnosis and/or cervical cancer prevention based on their diagnosis (Güvenç et al., 2013).

Taking into consideration that the CCS rates of women in Turkey are below the desired level and that the most important reason that women do not participate in CCS is lack of knowledge or awareness of CCS, studies that are designed to identify activities that will increase the participation of women in CCS are genuinely needed. Therefore, this study was designed to inform women about cervical cancer, the related risk factors, and the CCS program's role in both building awareness about the services of the national cancer screening program and removing perceived barriers using various interventions. The findings of this study are hoped to help increase the participation of women in CCS.

In view of the above, the primary purpose of this study was to assess the effectiveness of the three different interventions (training accompanied by a brochure, giving a brochure only, and giving an invitation without any information only) in terms of increasing awareness about cervical cancer, related risk factors, and the CCS as well as of removing the perceived barriers of women during home visits to increase the participation of women in national CCS and the uptake of CCS. Secondarily, the level of participation in CCS after invitation, health promotion perceptions, and providing knowledge related to cervical cancer and the Pap smear test was assessed.

## Methods

### Study Design and Participants

This interventional study was performed in the three Turkish provinces of Ankara, Malatya, and Trabzon between August 2011 and April 2012. Formal approvals were obtained from the Ministry of Health of Turkey (approval number: 10990335).

The target population consisted of women living in the regions served by the three KETEMs—Ankara Etlik Zübeyde Hanim Women's Hospital, Malatya State Hospital, and Trabzon Fatih State Hospital.

Home visits were made by the principal investigator to the women who met the inclusion criteria in each province. Those women who agreed to participate were randomly assigned to the three different interventions:

(i) One-on-one training accompanied by an educational brochure (education + brochure group)
(ii) An educational brochure only (brochure-only group)
(iii) An invitation only, without additional information (invitation-only group)

After the intervention, all participants were invited to the KETEMs for CCS. During the screening, all participants were informed about the role of the national CCS program in increasing awareness about services at the KETEMs. The principal investigator made only one home visit to each woman.

### Inclusion and Exclusion Criteria

The criteria for inclusion in this study were as follows: 30–65 years old (based on the age range of the national CCS); being or having been sexually active; able to speak, read, and understand the Turkish language; and providing informed consent.

Subjects were excluded from participation if they

- had gynecologic cancer;
- had received a hysterectomy;
- had received a Pap smear test during the previous year; and
- were pregnant or in the 3-month postpartum period.

### Study Sample and Intervention Groups

Convenience sampling was used to select the target provinces of Ankara, Malatya, and Trabzon, which were respectively located in the northern, eastern, and middle regions to provide representative social and cultural diversity.

After identifying the provinces, the sampling was made for groups that would be made available for home visits. Accordingly, a two-staged stratification sampling method was used to determine the study sample. A total of 134,704 women between the ages of 30–65 years resided in the three target provinces. Five hundred twenty women from each were calculated to be needed for the first-stage stratification. The total sample size was then stratified to each region for the second-stage stratification sampling. For this stage, it was assumed that the rate of Pap smear tests for each region was 25%, 5%, and 10%, respectively (these are the official rates estimated by the Republic of Turkey Ministry of Health's Cancer General Office). The sample size was calculated for each region as 96 in Ankara, 147 in Malatya, and 277 in Trabzon, with a 2.5% error rate and a 97.5% confidence interval. To stratify these women by province, Equation 1 was used:


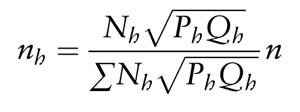


After determining the sample size, the contact information for the women was obtained from the KETEM registry. The contact information of the women was numbered and ordered and then was classified by block randomization into one of the three interventions. The investigator made one home visit to each randomly selected woman to determine if they met the eligibility criteria.

### Developing the Educational Brochure

An educational brochure was developed to invite the women for CCS at the KETEMs and to impart knowledge of and awareness about cervical cancer, CCS with Pap smear test, and the services provided at the KETEMs. In developing the educational brochure, the key concepts to be included to inform the participants about CCS were determined and then the content relevant to these concepts were developed. A comprehensive literature review was undertaken to design the brochure (Güvenç et al., 2013; Kög et al., 2012; Yücel et al., 2009). In addition, existing educational brochures were analyzed, and professionals in the field were consulted.

The tagline “Give yourself a chance, get regularly screened for cervical cancer” was used in the brochure to draw participants' attention. The educational brochure provided information related to “What is cervical cancer?”, “What are the causes and symptoms of cervical cancer?”, “What is a Pap smear test?”, “Why is the Pap smear test done?”, “When should the Pap smear test be done?”, and “Information about KETEMs.” In addition, the brochure invited women to participate in free CCS with Pap smear test at the KETEM in their area.

Content experts included a gynecologist and a senior nurse lecturer with expertise in women's health, both of whom were consulted during the prepublication evaluation of the brochure. These individuals participated in the evaluation of the brochures before printing. The brochure content was also reviewed by KETEM management for cultural sensitivity and education level appropriateness, and modifications were made based on KETEM's feedback.

The completed educational brochure was first administered by the investigator to 15 women as a pilot study to ascertain whether the content could be easily understood. The women in the pilot study were excluded from the main study.

### Instruments

Outcome data were obtained using the following surveys: “Participant Description Questionnaire,” “Knowledge Questionnaire about Cervical Cancer and Pap Smear Test,” and “Health Belief Model Scale for Cervical Cancer and the Pap Smear Test.”

#### Participant description questionnaire

The investigators developed this form based on a review of the literature. The form consists of questions that are used to determine the sociodemographic, obstetric, and gynecologic features of the women as well as Pap smear history and awareness of the available services at the KETEMs.

#### Knowledge questionnaire about cervical cancer and Pap smear test

This form consists of 10 questions that are designed to measure the basic knowledge related to cervical cancer and the Pap smear test. The questions were systemically prepared by the investigators using the same literature that was consulted in preparing the brochures. Participants were asked to mark the statements as either “true” or “false.” In the evaluation of the statements, a score of 1 was given for correct answers and 0 was given for incorrect answers. The range of total possible scores was 0–10, with higher scores indicating higher knowledge of cervical cancer and of the Pap smear test. The reliability of this questionnaire was calculated using the Kuder–Richardson 20 formula, which found a reliability coefficient of .80.

#### Health belief model scale for cervical cancer and the Pap smear test

The Health Belief Model (HBM) was developed by Güvenç, Akyüz, and Açikel in 2011 to measure the health beliefs of women in relation to cervical cancer and the Pap smear test. This scale includes 35 items, with all items scored on a 5-point Likert scale. The HBM includes five subscales: seriousness, susceptibility, Pap smear benefits, Pap smear barriers, and health motivation. The Pap smear barriers subscale is negatively associated and the other subscales are positively associated with screening behavior. As each scale was evaluated separately and not combined into a single total score, each participant obtained points for each subscale. In the original testing, Cronbach's alpha coefficients for the five subscales were .78, .78, .86, .82, and .62, respectively (Güvenç et al., 2011). In this study, Cronbach's alpha coefficients were calculated to be .84 for seriousness, .97 for susceptibility, .83 for Pap smear benefits, .56 for health motivation, and .79 for Pap smear barriers.

### Procedures and Data Collection

Consecutive home visits with the participants were made by the principal investigator. First, the compliance of women with the inclusion criteria was confirmed using the “Participant Description Questionnaire” during the home visit. Those who met the inclusion criteria for each group were then informed about the aim of the study and given the HBM and the “Knowledge Questionnaire about Cervical Cancer and Pap Smear Test” to complete. The participants in each intervention group were then invited to the KETEMs for CCS according to the interventions identified in the randomization table. In this regard:

(1) Brochure + education group: The principal investigator conducted one-on-one training regarding the importance of cervical cancer and the Pap smear test and distributed an educational brochure to the participants in this group. The training brochure was left with the women so that they could review it at their leisure. During the training session, the following information was explained to the women:

- Anatomical location of the cervix
- Prevalence, symptoms, and risk factors of cervical cancer
- Importance of the Pap smear test to the early diagnosis of cervical cancer
- Information about the Pap smear test
- How the Pap smear test is performed
- How frequently the Pap smear test should be performed
- Who should receive a Pap smear test
- Issues that need to be considered before getting a Pap smear test

(2) Brochure-only group: The participants in this group were asked to read the educational brochure.
(3) Invitation-only group: The participants in this group were invited to receive a CCS without receiving additional training or an educational brochure.

After the intervention, the women in each intervention group were informed about the CCS services that are regularly offered by KETEMs. Moreover, they were informed that these services are offered at no cost and without prior appointment or long wait times. In addition, they were told that treatment provided at the KETEMs is free when any problem is detected as a result of screening. The women were then invited to the KETEM in their region of residence for CCS.

**Figure 1.**
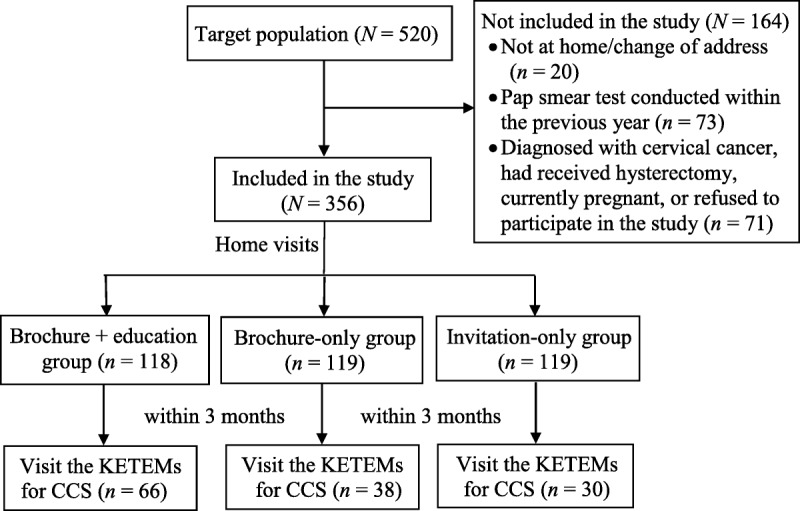
Flow chart of the study. CCS = cervical cancer screening; KETEMs = Cancer Early Diagnosis, Screening and Education Centers.

By the end of the study, 520 home visits were made. However, only 500 women were interviewed, as 20 women were unreachable because of a change of address. Of the women who were contacted (*n* = 500), 71 were excluded because of reasons including having undergone a hysterectomy, being currently pregnant, having had cervical cancer, and not agreeing to the interview. Thus, 429 were interviewed. However, 73 cases were not invited to the KETEM for CCS, as they had undergone a Pap smear test within the previous year. Therefore, the final study was conducted with 356 participants, including 118 women in the education + brochure group, 119 in the brochure-only group, and 119 in the invitation-only group (Figure 1).

#### Cervical cancer screening and form completion at Cancer Early Diagnosis, Screening and Education Centers

During the home visit, the participants were informed that they could visit the KETEM for CCS on any date they wished. After the data collection had been completed in a province, the participants were given a 3-month period in which to visit the KETEMs for CCS (see Table 1). In most studies, screening participation rates were calculated for a period ranging from 2 to 12 months after the invitation to receive a screening (Bowles et al., 2016; Kitchener et al., 2018; Taylor et al., 2002; Yücel et al., 2009).

**TABLE 1.**
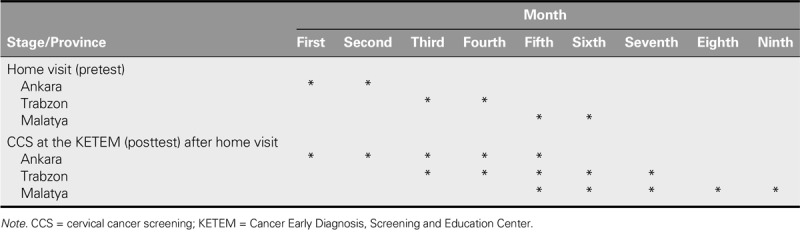
Time Points of Data Collection for Each Province

The Pap smear samples of the women in each group who appeared at the screening after invitation were collected by the KETEM staff, who, without prior knowledge of the participants' grouping, also administered the HBM and “Knowledge Questionnaire about Cervical Cancer and Pap Smear Test.” The completed data collection forms and cytology results of the women were sent by KETEM staff to the principal investigator via mail after the 3-month waiting period.

### Data Analysis

The study data were analyzed using SPSS Version 15.0 (SPSS Inc., Chicago, IL, USA). The distribution of the data was expressed as counts and percentages, and descriptive statistics were presented with arithmetical mean and standard deviation (*SD*). The conformity of the normal distribution of data was assessed using the one-sample Kolmogorov–Smirnov test, after which it was determined that the data were not indicators of a normal distribution. Because the data did not represent a normal distribution, the chi-square (χ^2^), Kruskal–Wallis, and Mann–Whitney *U* tests were used to determine the differences among the groups. The source of differences are typically investigated using the Bonferroni-corrected Mann–Whitney *U* test when a difference is found using Kruskal–Wallis analysis.

The effect of independent variables on the dependent variable was evaluated using single and multiple logistic regression analyses. The variables found to be significant in single-variable logistic regression were evaluated using the multiple backward logistic regression model.

Row or column percentages were provided in the tables, and their interpretations according to the position of the dependent variable were investigated. An explanation was provided under each table when line percentages were used. A *p* value of less than .05 was accepted as statistically significant.

## Results

In this study, 77.6% of the participants were between the ages of 30 and 49 years, with a mean age of 43.5 ± 8.13 years; 91.6% were married, 49% listed their highest level of education as primary school, and 89.3% were unemployed. No statistically significant differences in sociodemographic data were identified among the three groups.

Table 2 shows the distribution of the participants according to whether they participated in CCS after the interventions with home visit. The rates of participation for CCS with Pap smear test were 55.9%, 31.9%, and 25.2%, respectively, for the brochure + education, brochure-only, and invitation-only groups. The CCS rates were higher in the brochure + education group. The difference between the groups was highly significant (*p* < .001; Table 2).

**TABLE 2.**
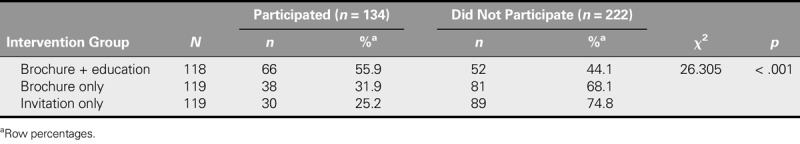
The Distribution of Participants According to Participation in Cervical Cancer Screening Status After the Interventions With Home Visit (*N* = 356)

Table 3 presents the distribution of the mean preintervention and postintervention knowledge scores, HBM subscores, and score differences among the intervention groups. A statistically significant difference was identified between the intervention groups in terms of preintervention knowledge scores (*p* < .05). The source of the difference was investigated using the Bonferroni-corrected Mann–Whitney *U* test. The preintervention knowledge scores of the brochure + education group was found to be lower than the invitation-only group (*z* = 3.610, *p* < .001).

**TABLE 3.**
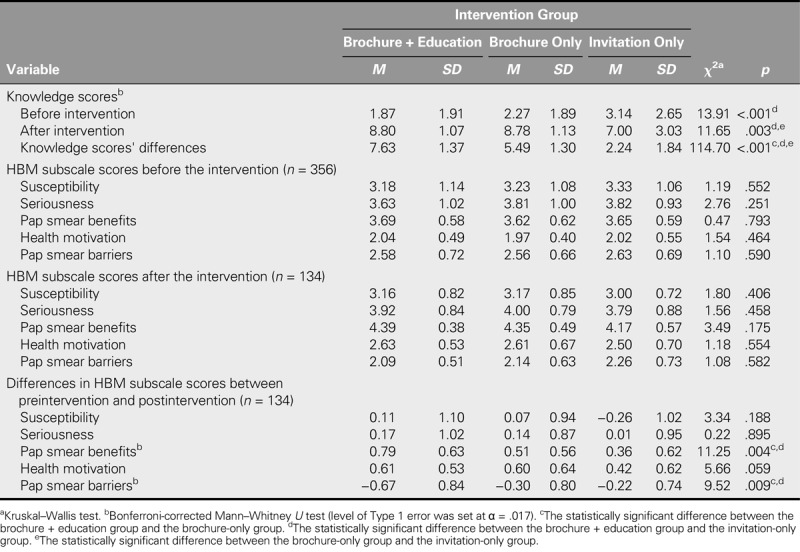
The Distribution of Mean Preintervention and Postintervention Knowledge Scores, Health Belief Model (HBM) Subscores, and Score Differences of Participants, by Intervention Group

Furthermore, a statistically significant difference was found between the intervention groups in terms of postintervention knowledge scores (*p* < .05). According to the pairwise comparisons, which were conducted to identify intervention-related differences between the groups, the postintervention knowledge scores in the brochure + education group were higher than in the invitation-only group (*z* = 3.116, *p* = .002). In addition, the postintervention knowledge scores in the brochure-only group were higher than in the invitation-only group (*z* = 2.878, *p* = .004).

When the change between the preintervention and postintervention knowledge was assessed by group, the difference in the brochure + education group was higher than in both the brochure-only group (*z* = 7.099, *p* < .001) and the invitation-only group (*z* = 8.928, *p* < .001). Furthermore, the knowledge increase in the brochure-only group was higher than in the invitation-only group (*z* = 7.153, *p* < .001). All of these differences were highly significant (*p* < .001).

No statistically significant difference was found among the intervention groups in terms of the preintervention and postintervention HBM subscale scores (*p* > .05).

The difference in the changes in score between the preintervention and postintervention HBM benefits and Pap smear barriers subscales was found to be significant across the intervention groups. The intergroup differences were found primarily between the brochure + education group and the brochure-only group (*z* = 2.455, *p* = .014) and the invitation-only group (*z* = 3.040, *p* = .002) in terms of the Pap smear benefits subscale scores and between the brochure + education group and brochure-only group (*z* = 2.442, *p* = .015) and between the brochure + education group and invitation-only group (*z* = 2.712, *p* = .007) in terms of the Pap smear barriers subscale scores. The perceived Pap smear benefits and the perceived Pap smear barriers among the brochure + education group participants respectively increased and decreased more than among their invitation-only group peers (*p* < .05; Table 3).

Univariate and multiple logistic regression analyses were performed to examine in a multifactorial manner the factors affecting postintervention CCS rates as well as to determine the relationship between these factors. Univariate logistic regression analysis found that the sociodemographic factors of the participants (age, education, occupational status), number of pregnancies, age at first marriage and at first childbirth, menopausal status, preintervention awareness of KETEM, family history of cancer, and health beliefs (except Pap smear benefits and health motivation) had no effect on screening rates. However, multiple logistic regression analysis showed that being invited with a brochure + education and a high knowledge score had a significant influence on participation in CCS after the intervention with home visit. The participation rate in CCS of the participants in the brochure + education group was 3.05 times higher than among those participants who were only given a brochure and 5.46 times higher than among those participants who were only given a personal invitation.

A positive relationship was found between the postintervention knowledge score and participation in CCS. Each unit increase in knowledge score was associated with an 0.83 times increase in the likelihood of participation in CCS (Table 4).

**TABLE 4.**
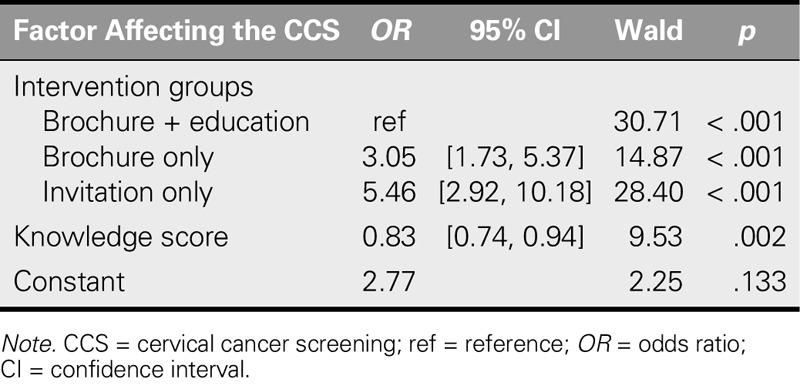
The Multivariable Investigation of the Factors Affecting Participation in Cervical Cancer Screening After the Interventions and the Results of Multiple Logistic Regression Analysis

## Discussion

In Turkey, as in many developing countries, the CCS rate is not yet at the desired level (Babacan Gümüş & Çam, 2011; Güvenç et al., 2013; Uluocak & Bekar, 2012; Yücel et al., 2009). This study assessed the effectiveness of three different interventions in increasing participation in the national CCS.

The sociodemographic characteristics of the women in the three groups were similar in this study, which is important in terms of showing the similarity among the groups being compared. In this study, 55.9% of the participants in the brochure + education group, 31.9% in the brochure-only group, and 25.2% in the invitation-only group went to KETEMs after the intervention and home visit to receive a CCS with Pap smear test. The participants in the brochure + education group had a higher Pap smear test rate than the those in the other intervention groups (Table 2). This result supports educational training accompanied by a brochure during home visits as the most effective intervention for increasing the participation of women in CCS. In a study conducted by KETEM staff in the same region in which our study was conducted, 10,963 women were invited (through letters and phone calls) for cervical and breast cancer screenings between 2008 and 2010. Over the course of the study, 1,478 women (13.5%) visited the KETEM for screening (Kög et al., 2012). In another study, which was conducted to increase the participation in CCS of Chinese women living in North America, Taylor et al. left educational materials during home visits with one group of women who had not had a Pap smear test, sent educational materials by mail to another group, and implemented no intervention to the third group. They found women in the educational materials + home visit group to have a higher participation rate in CCS than their peers in the other two groups at 6 months postintervention. They also found providing the information during home visits to be an effective approach to educate women about participating in cancer screening programs and to increase participation in these programs (Taylor et al., 2002). Providing instructor-based training and educational materials has been found to be the most effective method to increase the participation of women in CCS in other similar studies (Güvenç et al., 2013; Lam et al., 2003; Nguyen et al., 2006; Yücel et al., 2009). On the basis of this and previous studies, screening programs alone are not sufficient to encourage the participation of women in CCS. Therefore, the organization and substructure of the health system should be reconstituted accordingly, and a service model for informing women about screening programs as well as for providing training and raising awareness should be developed.

In addition, the comparative effectiveness of providing a brochure + education and only giving a brochure was tested in terms of increasing knowledge of cervical cancer and the Pap smear test. Although the mean pretest knowledge score of the brochure + education group was the lowest of the three groups, the mean score for this group and the brochure-only group both significantly increased after the intervention. The highest difference in preintervention and postintervention knowledge scores was attained by the brochure + education group, whereas statistically significant increases were also found for the brochure-only and invitation-only groups (Table 3). Considering that the postintervention screening rate was higher in the brochure + education group, it may be argued that knowledge of cervical cancer and the Pap smear test affects participation rates in CCS. On the other hand, the presence of increased knowledge in the group given only a brochure may also indicate that the women read the brochure and followed its recommendations. Similarly, other studies that were conducted to increase the participation of women in CCS indicate that knowledge scores increase significantly after participants receive cervical cancer and the Pap smear test training (Damiani et al., 2015; Ferris, Hupman, Waller, Cudnik, & Watkins, 2009; Güvenç et al., 2013; Visanuyothin et al., 2015).

The cervical-cancer and Pap-smear-test-related health beliefs of women, including perceived susceptibility, perceived seriousness, and perceived barriers, strongly influence screening behavior and participation in CCS (Güvenç et al., 2011). In this study, the health beliefs of women related to cervical cancer and the Pap smear test were evaluated to determine the perceived barriers related to screening behavior. The preintervention mean HBM subscale scores of the participants in all of the three intervention groups were similar in this study, which is important in terms of showing the pretest similarity among the groups. Moreover, the HBM subscale scores of the three groups were similar after the intervention. However, when evaluated in terms of the changes between the preintervention and postintervention HBM subscores by intervention group, the participants in the brochure + education group who received Pap smear tests after being invited reflected significantly improved Pap smear benefit perceptions and significantly lower perceived barriers to receiving Pap smears than their peers in the other intervention groups. Using different intervention methods did not indicate a change in the seriousness, susceptibility, or health motivation perceptions of the participants (Table 3). A similar study conducted to increase the rate of screening showed that the Pap smear benefit perceptions of the group provided training on cervical cancer and the Pap smear test were significantly higher and that their Pap smear barrier perceptions were significantly lower (Park, Chang, & Chung, 2005). Similarly, another study that was conducted to increase the participation in CCS of women in Turkey reported that the perceived barriers of women toward the Pap smear test decreased after educational interventions (Güvenç et al., 2013). Another related study reported that the Pap smear barrier perceptions of women decreased after receiving interventions to increase participation in CCS (Boonpongmanee & Jittanoon, 2007). In this regard, it is likely that interventions to increase participation in CCS lead to a better understanding of the importance and benefits of the Pap smear test, potentially leading to earlier diagnoses of cervical cancer and reduced perceived barriers to receiving the test.

This study shows interventions and knowledge score to be effective in increasing the uptake of CCS after home visits, where the interventions that were used to increase the participation of women in CCS programs were evaluated. The most effective intervention for increasing participation in CCS was invitation using a brochure + education. The CCS participation rate among the participants in the brochure + education group was 3.05 times higher than among those who were only given a brochure and 5.46 times higher than among those who were only verbally invited. Moreover, a positive association was observed between knowledge scores and probability of CCS participation (Table 4). Similarly, other studies have shown that home visits and one-on-one training administered by an instructor are effective in encouraging the participation of women in national cervical cancer control programs and in increasing their knowledge on the subject (Albada, Ausems, Bensing, & van Dulmen, 2009; Spadea, Bellini, Kunst, Stirbu, & Costa, 2010; Studts et al., 2012).

### Implications for Clinical Practice and Research

Women's knowledge of cervical cancer and CCS after education and raised awareness plays a significant role in influencing their decision to be screened. The use of training accompanied by a brochure during home visits, the active participation of nurses during screenings, and the distribution of necessary resources to current health organizations (qualified staff, vehicles to conduct home visits, etc.) are highly recommended to increase the participation of women in CCS, the uptake of cervical screening, and related awareness. Furthermore, all healthcare professionals should be educated regarding the current CCS recommendations for women and be made aware of available community-based screening programs.

In this study, women with high perceived barriers related to the Pap smear test (e.g., embarrassment about the gynecologic examination, fatalistic beliefs about healthcare behaviors) tended not to participate in CCS. Therefore, interventions that are aimed to increase participation in CCS should focus on the health beliefs and cultural norms that are specific to the women in each target community.

### Conclusions

This study found providing training accompanied by a brochure to be the most effective intervention in terms of increasing participation in CCS. These results support that interventions that are conducted to increase knowledge and awareness of cervical cancer are effective in encouraging participation in CCS.

This study is affected by several limitations. First, the Cronbach's alpha value of one of the HBM subscales was lower than .70. Second, this study was limited to three provinces from three regions of Turkey. Moreover, only women currently residing in the main city in these provinces were interviewed. The effectiveness of the recommended methods in encouraging the participation of women in CCS across a broad range of areas (e.g., both urban and rural areas) should be evaluated in future studies.
